# Does Hypoxia Cause Carcinogenic Iron Accumulation in Alcoholic Liver Disease (ALD)?

**DOI:** 10.3390/cancers9110145

**Published:** 2017-10-25

**Authors:** Inês Silva, Vanessa Rausch, Helmut-Karl Seitz, Sebastian Mueller

**Affiliations:** Center for Alcohol Research, University of Heidelberg and Salem Medical Center, 69120 Heidelberg, Germany; vanessa.rausch@uni-heidelberg.de (V.R.); helmut_karl.seitz@urz.uni-heidelberg.de (H.-K.S.)

**Keywords:** ALD, HCC, hepatic iron overload, hepcidin, hydrogen peroxide, hypoxia, GOX/CAT system, NOX4, oxidative stress

## Abstract

Alcoholic liver disease (ALD) is a leading health risk worldwide. Hepatic iron overload is frequently observed in ALD patients and it is an important and independent factor for disease progression, survival, and the development of primary liver cancer (HCC). At a systemic level, iron homeostasis is controlled by the liver-secreted hormone hepcidin. Hepcidin regulation is complex and still not completely understood. It is modulated by many pathophysiological conditions associated with ALD, such as inflammation, anemia, oxidative stress/H_2_O_2,_ or hypoxia. Namely, the data on hypoxia-signaling of hepcidin are conflicting, which seems to be mainly due to interpretational limitations of in vivo data and methodological challenges. Hence, it is often overlooked that hepcidin-secreting hepatocytes are physiologically exposed to 2–7% oxygen, and that key oxygen species such as H_2_O_2_ act as signaling messengers in such a hypoxic environment. Indeed, with the recently introduced glucose oxidase/catalase (GOX/CAT) system it has been possible to independently study hypoxia and H_2_O_2_ signaling. First preliminary data indicate that hypoxia enhances H_2_O_2_-mediated induction of hepcidin, pointing towards oxidases such as NADPH oxidase 4 (NOX4). We here review and discuss novel concepts of hypoxia signaling that could help to better understand hepcidin-associated iron overload in ALD.

## 1. Introduction

In high-income countries, alcohol consumption is the sixth leading cause of premature death. While the liver is the major target organ of alcohol consumption, liver cirrhosis and cirrhosis-associated hepatocellular carcinoma (HCC) are the main causes of alcohol-attributed mortality [1,2]. The term alcoholic liver disease (ALD) refers to the broad spectrum of liver damage caused by excessive alcohol intake, ranging from benign steatosis to steatohepatitis and cirrhosis.

Alcohol-mediated carcinogenesis is complex and still poorly understood. The total comprehension of carcinogenesis by alcohol is hampered by the lack of reliable models that can recapitulate human alcohol metabolism. Notably, commonly used rodent models show significant differences with regard to alcohol metabolism, sensitivity, and toxicity, leading to a less severe phenotype [3]. In fact, the end liver stage cannot be induced solely by ethanol in rats and mice, but only in combination with other toxicity models. However, one of the key features of ALD is the accumulation of carcinogenic iron in the liver. Free iron in the presence of both enhanced oxidative stress and hypoxia can be highly tumorigenic, eventually leading to HCC [4].

The underlying molecular mechanisms of differential iron deposition, however, remain unclear, as is the role of hepcidin. For instance, acute exposure to alcohol drastically suppresses hepcidin, which could explain the ultimate accumulation of hepatic iron [5]. However, no significant hepcidin differences have been observed between ALD patients with high and low iron. Moreover, iron sensing by hepcidin is still not clear. It is also unclear as to why hepcidin responds differently in isolated liver cells as compared to in vivo conditions. In addition, conflicting data has been published regarding the impact of liver hypoxia on iron metabolism, as well as the effect of hypoxia in combination with oxidative stress, mainly H_2_O_2_, which resembles a typical environment found in ALD. In this review we will briefly summarize the recent knowledge on iron accumulation in ALD, with a focus on hypoxia and H_2_O_2_ in the regulation of hepcidin.

## 2. Hepatic Iron Overload in ALD and Carcinogenesis

**Figure 1:**
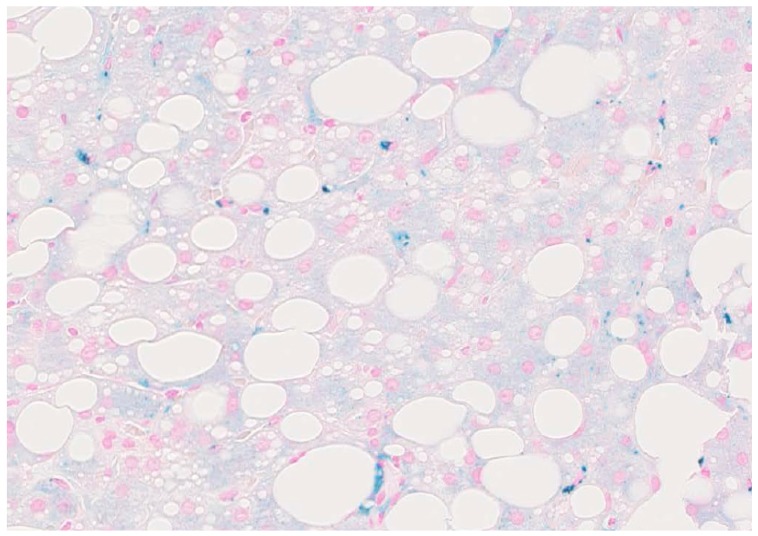
Histopathology from a patient with alcoholic liver disease (ALD) shows pathological iron accumulation. Prussian blue stain from a liver biopsy of a 47-year old male patient with clear features of ALD (macrovesicular lipid droplets) and iron deposits (blue stain in hepatocytes and Kupffer cells).

Hepatic iron accumulation and chronic alcohol consumption have long been linked (Figure 1) [6]. In ALD patients, iron has been identified as independent risk factor for survival and HCC development [7]. Moreover, the correlation between excessive alcohol intake and reduced survival in hemochromatosis patients has been established as well [8].

**Figure 2:**
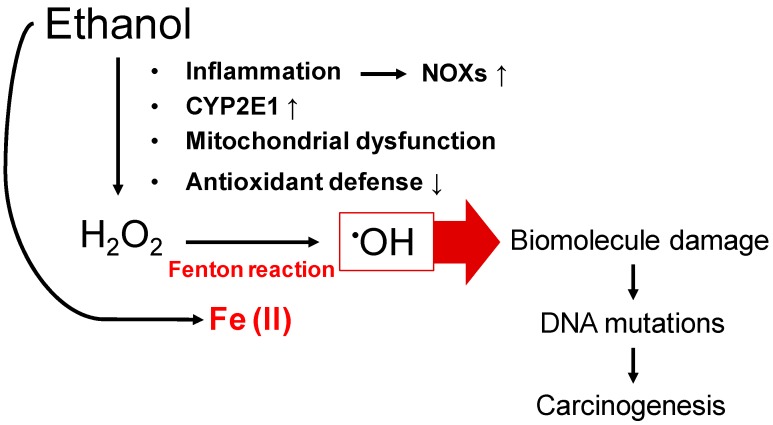
Schematic overview of current knowledge of mechanisms leading to ethanol and iron-induced carcinogenesis. CYP2E1: Cytochrome P450 2E1; NOXs: NADPH oxidases.

The carcinogenic potential of tissue iron accumulation is highly attributed to Fenton-like reactions which occur in the presence of ferrous iron and H_2_O_2_, yielding to highly reactive hydroxyl radicals (Figure 2) [3]. Several enzymes are able to generate H_2_O_2_, predominantly during the inflammation typically observed in ALD, however cytochrome P450 2E1 (CYP2E1) and its induction by ethanol is regarded as a key player in alcohol-mediated reactive oxygen species (ROS) production [2]. Thus, chronic alcohol consumption results in an up to 20-fold increase in CYP2E1 expression, leading to the release of ROS such as H_2_O_2_, hydroxyl, superoxide and hydroxyethyl radicals via not completely understood mechanisms [9]. Furthermore, ethanol-induced oxidative stress is also the result of an impaired antioxidant defense mechanisms as well as impaired synthesis of mitochondria-encoded constituents of the respiratory chain, particularly cytochrome b and the production of ROS by leakage from complex I and III of the respiratory chain [10]. In addition, activated phagocytes also contribute to ROS [10]. Excessive iron accumulates in lysosomes, originating from auto-phagocytosed ferritin and hemosiderin. It often leads to fragile membranes via lipid peroxidation and subsequent lysosomal dysfunction with the loss of free iron into the cytoplasm [11]. The iron-associated toxicity is due to mutagenic effects of free radicals resulting in DNA damage and structural alteration of lipids and proteins. An example is the cleavage of lipid hydroperoxides, resulting in aldehydes, such as malondialdehyde (MDA) and 4-hydroxynonenal (4-HNE), which can react with the ε-NH_2_ group of lysine and histidine residues [12] and DNA bases [13] forming extremely mutagenic adducts. Indeed, these adducts can specifically target the p53 tumor suppressor gene, conferring apoptotic resistance to the cells [14]. Therefore, 4-HNE has been used as an indicator of radical-mediated cellular damaged and oxidized hepatocytes. It is also significantly correlated with iron deposition in the liver [15]. Recent in vitro studies indicate that mitophagy, the selective clearance of damaged mitochondria by autophagy in the liver, may also play a role in mediating hepatocyte apoptosis [16]. Acute and chronic ethanol treatment in various animal models results in enhanced mitophagy which is associated with mitochondrial translocation of cytosolic Parkin (an E3 ubiquitin ligase maintaining mitochondrial homeostasis) caused by oxidative mitochondrial DNA damage. Parkin also co-localizes with accumulated 8-Hydroxydesoxyguanosin a marker of oxidative mitochondrial DNA damage in ethanol-exposed hepatocytes which may be a stimulus for DNA repair and the prevention of carcinogenesis [17]. For these reasons and because the mitochondria is the main target organelle for alcohol toxicity, modulation of Parkin translocation may be a new therapeutic option in ALD in the future after further investigation.

Taken together, the actual role of iron (in particular non-protein iron complexes) and how alcohol favors iron accumulation is still under investigation. It has been proposed that oxidative modifications of the cytosolic iron regulatory protein 1 (IRP1) might cause dysregulations in ferritin synthesis and induce the synthesis of the transferrin receptor, eventually leading to increased iron uptake combined with impaired liver storage capability [10]. Overall, there is much evidence that iron overload and oxidative stress in ALD is tightly linked with progression to hepatocellular damage, liver-related death, and cancerogenesis.

## 3. Control of Iron Homeostasis

**Figure 3:**
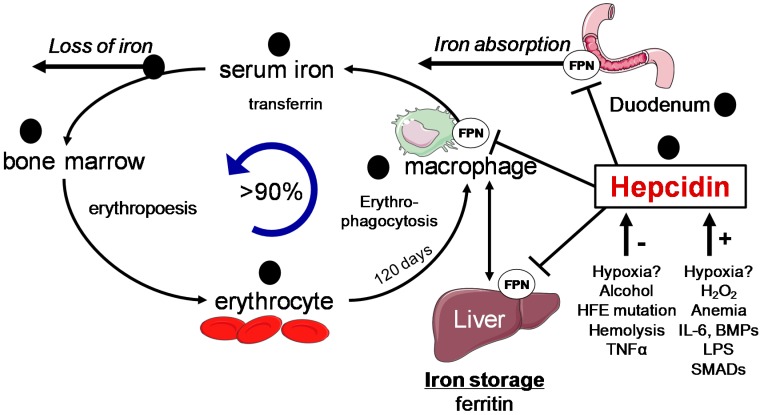
Iron homeostasis and utilization in the body (adapted from [3]). Dietary iron is absorbed in the duodenum and binds to transferrin. Iron is delivered to the bone marrow for erythropoiesis, senescent erythrocytes are phagocytosed by the macrophages, and iron is recycled for heme synthesis. Excess iron is stored in ferritin in the liver. Regulation of iron metabolism by hepcidin and factors which influence hepcidin expression are also shown. Black circles indicate potential sites of alcohol interference. BMP: Bone morphogenic protein; FPN: Ferroportin; HFE: hemochromatosis protein; IL: interleukin; LPS: lipopolysaccharide; SMAD: Suppressor of mothers against decapentaplegic; TNF: Tumor necrosis factor.

In the last decades, an enormous progress has been made to better comprehend the molecular mechanisms of iron regulation and homeostasis at the systemic and the cellular level, however several aspects remain unclear [18,19]. In humans, most of the body’s iron is found in the oxygen-carrying hemoglobin of erythrocytes. The remaining iron is stored in hepatocytes and in reticuloendothelial macrophages in the form of ferritin. The liver represents an important reservoir of excess iron, while macrophages phagocyte the senescent erythrocytes and load the iron from hemoglobin onto transferrin for iron recycling [20]. Dietary uptake of iron is carried out by duodenal enterocytes and its efficient control is curtailed for maintenance of the homeostasis, since iron can also be passively lost from regular sloughing of the mucosa and skin or during bleeding (Figure 3) [18]. Figure 3 also highlights multiple sides of impaired iron regulation by alcohol [3].

### 3.1. Cellular Regulation of Iron

Erythroid cells, as with all other cell types, depend on the delivery of iron via the iron carrier serum transferrin (Tf), a glycoprotein with two affinity sites for ferric iron. Diferric Tf binds with high affinity to cell surface transferrin receptor 1 (TfR1) and with lower affinity to transferrin receptor 2 (TfR2) [21]. Despite their homology, TfR2 does not significantly contribute to iron import and it is regarded as a sensor of Tf saturation. Genetic deletion of TfR1 in mice demonstrates its endocytotic role and ability to import iron into several cell types [22]. In non-erythroid cells, iron is safely stored in ferritin complexes or can be incorporated into hemoglobin of erythrocytes, being later reused for various synthesis pathways [23]. Ferric iron stored in ferritin complexes (non-toxic form) must be subsequently released for biological use via lysosomal degradation of ferritin [24]. This mechanism of autophagy dominates during iron deficiency and is mediated by the nuclear receptor coactivator 4 (NCOA4) [25]. It has been described that NCOA4 interacts with ferritin heavy chain targeting ferritin for degradation. In iron overloaded cells, NCOA4 expression decreases, leading to suppression of ferritin autophagy [26]. A recent study described the retention of iron within ferritin in NCOA4 knockout (KO) mice, which led to iron-deficiency anemia, highlighting the important role ferritin autophagy mechanism on cellular and systemic iron homeostasis [27].

At the cellular level, central regulators of iron homeostasis are controlled post-transcriptionally by iron responsive proteins (IRP1 and IRP2) which are able to bind to the iron-responsive elements (IREs) of the RNA encoding for various iron-related proteins. While binding to the IRE located in the 5’ untranslated region of the mRNA results in a translational inhibition, the binding of IRPs to the 3’ untranslated region stabilizes and protects the transcripts from degradation. In particular, IRPs can cause upregulation of TfR1 or suppress the translation of mRNA encoding other proteins involved in iron metabolism, such as ferritins or ferroportin (FPN) [28]. During cellular iron deficiency, IRPs are in the active form (apo-IRP) and this results in TfR1 induction stimulating the acquisition of iron from plasma Tf. In contrast, to counteract iron overload, IRPs become inactive (holo-IRP) for IRE binding, leading to degradation of TfR1 mRNA and translation of ferritin mRNA [29].

The interactions of IRE–IRP allow an autonomous independent control of iron homeostasis for individual cells, however this network can be also overwritten by additional controls mechanisms. Namely, TfR1 expression is also regulated at the transcriptional and translational level [30,31]. Interestingly, IRP1 is regulated in a complex manner by various ROS linking iron homeostasis to oxygen metabolism [32,33,34].

### 3.2. Systemic Iron Control by Hepcidin

Systemically, iron is mainly controlled by the hormone hepcidin (Figure 3). This 25 amino-acid peptide is secreted form the liver and is primarily expressed in hepatocytes, although low expression levels have been also reported in macrophages [35,36]. By binding to the unique iron exporter, ferroportin (FPN), hepcidin efficiently inhibits duodenal iron absorption, iron recycling from macrophages, and iron mobilization from hepatic stores. Hepcidin blocks the iron efflux into the plasma by binding FPN and consequently inducing the phosphorylation, internalization, and lysosomal degradation of the complex by the proteasome [37]. Deletion of hepcidin in mice or hepcidin deficiency in humans results in severe hepatic iron overload, increased serum iron levels, and loss of iron in macrophage stores, caused by hyperabsorption of iron [38]. In contrast, transgenic overexpression of hepcidin causes decreased serum iron leading to anemia by blocking the iron absorption in enterocytes and the release of iron from the hepatic stores [39,40].

In mice models, acute exposure to ethanol rapidly suppresses hepcidin [41]. The exact mechanisms of how alcohol per se regulates hepcidin expression in the liver are still unclear. First, it has been shown that ethanol downregulates hepcidin promoter activity and the DNA binding activity of CCAAT/enhancer-binding protein **α** (C/EBP**α**) but not **β** in mice [5], leading to downregulation of hepcidin gene transcription, thereby increasing duodenal iron transport. Recently it has been shown that alcohol exerted different effects on transforming growth factor (TGF-β)-mediated activation of suppressor of mothers against decapentaplegic (SMAD) 2 and bone morphogenetic protein (BMP)-mediated SMAD1 and SMAD5 activation [42]. Findings suggest the simultaneous inhibition of BMP-mediated SMAD activation and stimulation of TGF-β-mediated SMAD activation by alcohol in the involvement of hepcidin suppression in vivo. This fact, together with the conjugation of multiple signals acting in concert in regulating hepcidin, makes research on hepcidin regulation extremely complex. In patients with ALD, significantly suppressed hepcidin mRNA levels were found in the liver which, however, was restricted to patients with preserved hepatic function [43]. More recently, Nahon and colleagues assessed the influence of serum hepcidin levels on the long-term survival of patients with alcoholic cirrhosis. The risk of developing HCC was higher in patients with lowered circulating hepcidin concentrations, which was suggested to be independently associated with death [44]. In addition, we could recently demonstrate that hepcidin was at the very least not adequately upregulated in those patients manifesting a histological iron overload [3,45].

## 4. Transcriptional Regulation of Hepcidin

The levels of circulating hepcidin are mostly controlled at the transcriptional level. Hepcidin promoter activity can be induced by iron signals, including serum iron concentrations and liver stores, or by inflammatory signals and suppressed during increased erythropoietic activity. In general, the transcriptional control of hepcidin by iron occurs via BMP/SMAD pathway. High circulating concentrations of transferrin-bound iron (Tf-Fe) are the extracellular signal for transcriptional induction of hepcidin [46]. Tf-Fe modulates the interaction between the transferrin receptors (TFRs) 1 and 2 and the hemochromatosis protein (HFE) by inhibiting the binding of HFE to TFR1. Consequently, HFE stabilizes activin receptor-like kinase 3 (ALK3), which activates BMP/SMAD signaling cascade [47]. Increased Tf-Fe concentrations can also promote the association between HFE and TFR2 that can further form a membrane complex with the BMP the co-receptor hemojuvelin (HJV), promoting hepcidin transcription via the BMP/SMAD pathway [48]. Besides iron, inflammatory signals such as TGF-β, activin B, and BMPs are also inductors of BMP/SMAD signaling, while matripase-2 and furin act as suppressors by cleavage of the cell surface hemojuvelin (HJV) protein [49,50,51,52,53].

Increased erythropoiesis, caused by exposure to high altitude, anemia, or other physiological conditions, is so far described as the major inhibitory stimuli of hepcidin synthesis. Increased erythropoietin (EPO) release by the kidney is the major feature of erythropoiesis, being the proposed critical factor for erythropoiesis-mediated downregulation of hepcidin [54]. Despite many efforts over the last decade, there are still many open questions on how EPO suppresses hepcidin. Years ago, several studies have associated the suppression of hepcidin with two erythroid regulators (growth differentiation factor 15 (GDF15) and twisted gastrulation BMP signaling modulator 1 (TWSG1)), which are normally increased during erythropoiesis. However, the direct link between these proteins and hepcidin regulation is still missing [46]. A more recent study has identified another erythroid regulator as part of the hepcidin–EPO axis, called erythroferrone (ERFE) [55,56].

The induction of hepcidin during inflammation or infection constitutes an important evolutionary conserved mechanism known as “anemia of chronic disease”. This mechanism describes a host defense response against invading extracellular pathogens, in which interleukin-6 (IL-6) plays a key role as a major upstream regulator via the signal transducer and activator of transcription 3 (STAT3) pathway, leading to hepcidin induction [57]. Recently, H_2_O_2_ has been suggested as an additional important inflammatory cofactor and second messenger capable of upregulating hepcidin by activation of the STAT3 signaling cascade [58]. In particular, hepcidin can be strongly induced by exposing hepatoma cells to sustained H_2_O_2_ concentrations similar to those released by inflammatory cells [58]. Subsequent studies have confirmed the role of STAT3 in H_2_O_2_-mediated hepcidin induction [59,60]. Other studies reported contrary findings and demonstrated a suppression of hepcidin in alcohol-fed mice by ROS [5]. However, no mechanistic details were provided, and we had earlier shown that the concentration of peroxide is crucial for hepcidin transcription [58]. While low levels induce hepcidin, toxic levels drastically block hepcidin, most likely through unspecific inhibition of the transcription machinery.

In the context of ALD, H_2_O_2_ has been shown to have a complex, concentration-dependent and bivalent action on hepcidin. Since ethanol metabolism strongly affects hepatic oxygen homeostasis, liver hypoxia is thought to have an important impact on hepcidin regulation in vivo and in vitro [61].

## 5. Regulation of Hepcidin by Hypoxia

The hypoxic response is mainly controlled by hypoxia-inducible factors (HIFs), which comprise an oxygen-dependent α subunit (HIFα) and the constitutively expressed β subunit (HIFβ). Under adequate oxygen levels, prolyl hydroxylase domain-enzymes (PHDs) allow the binding of HIFα to the Von Hippel-Lindau protein (VHL) leading to proteasomal degradation of HIFα. In contrast, decreased oxygen levels cause stabilization of HIFα by inhibition of PHDs and consequent block of VHL action [62].

Over the last decade, the role of hypoxia and HIFs in hepcidin regulation has been extensively investigated and the first reports in this field have shown the in vitro and in vivo inhibition of hepcidin at the mRNA level under hypoxic conditions [63,64]. In order to elucidate the molecular mechanisms involved in hypoxia-mediated hepcidin regulation, several authors have used HIF and VHL KO mice models to dissect the role of HIFs in controlling hepcidin promoter activity. A genetic study with iron deficient and VHL KO mice in conjugation with transcriptional assays have suggested that HIF1 stabilization downregulates hepcidin, however, HIF1 alone was insufficient to explain hepcidin suppression under hypoxia. The same study suggested the direct suppression of hepcidin via a putative hypoxia response element located in the hepcidin promoter [65] but no further evidence could be provided until now. Moreover, subsequent reports suggested that HIF does not directly suppress the transcription of hepcidin [66,67]. Most recently, in vivo studies have shown that HIF-mediated suppression of hepcidin occurs indirectly through EPO-induced erythropoiesis, highlighting the contribution of HIF2 to hepcidin regulation [68,69]. Up to now, human studies have been published investigating the circulating hepcidin levels during the exposure to hypobaric hypoxia in conditions simulating high altitude [70,71]. The role of a new factor, the platelet-derived growth factor BB (PDGF-BB), was recently described in humans and mice. The circulating concentration of PDGF-BB highly correlates with hepcidin but not with other parameters. In addition, PDGF-BB could suppress hepcidin mRNA in hepatoma cells and primary hepatocytes [72].

While these reports clarified the regulation of hepcidin by systemic hypoxia, in which renal EPO release induces erythropoiesis, depleting the serum iron, the in vivo response to specific liver hypoxia is still lacking. The control of hepcidin by hepatospecific hypoxia has been extensively descried in vitro, resulting in multiple contradictory findings (Table 1). In general, it has been suggested that hypoxia induces the expression of hepcidin suppressors. Silvestri and colleagues identified HIF1α as a downregulator of hepcidin transcription, through induction of furin [73]. Another study has identified increased expression of matriptase-2 in conditions of severe hypoxia. The authors emphasized the role of HIF1α and HIF2α for the hypoxia-mediated suppression of hepcidin, while subsequent experiments performed in primary mouse hepatocytes discarded HIF2α as hepcidin regulator [69,74]. At the same time, an in vivo study reported the ethanol-mediated hepcidin suppression by stabilization of HIF in the liver. The direct role of HIF on hepcidin regulation was again suggested after experiments performed in VHL KO mice [75]. In contrast to these findings, the upregulation of hepcidin under hypoxia was also reported in vitro in hepatoma cell lines [67,76]. Moreover, downregulation of hepcidin could be obtained by co-culturing hepatoma cells with macrophages, however, the co-culture ratio used in this study did not reflect physiological conditions [76].

In summary, conflicting and partly confusing data have been represented so far with regard to hepcidin regulation by hypoxia. We believe that these contradictions are mainly due to limitations in the interpretation of in vivo models of hypoxia due to many adaptive responses at various regulatory levels and to methodological limitations that will be discussed in the next chapter.

## 6. Hepatic Oxygen Levels and Methodological Challenges to Studying Hypoxia

Many studies are typically performed under aerobic conditions of 21% oxygen. However, under physiological conditions, the average intracellular oxygen tensions in the liver are between 45 and 50 mm Hg (6–7% O_2_) in the periportal area and 15 to 20 mm Hg (2–3% O_2_) in perivenous tissue [78]. During acute or chronic alcohol consumption, liver oxygen levels are even lower due to the increased hepatic metabolic activity as well as alterations in hepatic blood flow caused directly by alcohol [79,80]. The diverse effects of acute and chronic ethanol exposure on cellular signaling, cellular metabolism, and organ physiology have been extensively reviewed elsewhere [81] as has the development of hypoxia in alcohol-exposed liver [82]. In brief, acute alcohol causes a rapid increase in liver metabolism, including the rapid activation of alcohol detoxifying enzymes, e.g. CYP2E1, and an increase in hepatic oxygen consumption, as shown by enhanced staining of pimonidazole, a hypoxia-specific marker [83]. This is accompanied by stabilization of HIF in the liver and increased hepatic steatosis in the setting of alcohol [84]. Recent gene array data from ethanol and pair-fed mice showed an upregulation of multiple genes involved in the glycolytic pathway as well as in lipid metabolism [85] with most of the genes being HIF targets and thereby regulated, suggesting a complex regulatory circuit.

Cells respond both to oxygen levels and oxygen changes, which will be strongly affected by the hypoxic system used in in vitro studies. Thus, mostly hypoxia chambers are commonly explored. Unfortunately, due to diffusion barriers of the culture medium, this system presents significant delays to reach oxygen equilibration on the bottom of the cell culture dishes. The definitive and constant hypoxia levels are typically achieved after 4 to 24 h, which fail to be reproduced in an in vivo situation [86]. The recently introduced glucose oxidase/catalase (GOX/CAT) system allows rapid depletion of the oxygen in the culture medium and a stable maintenance of hypoxia along the time [87]. This system has already been successfully explored in hypoxia studies [86,88].

Another important problem is the interpretation and, most likely, overestimation of in vivo models. Several genetically modified mouse models, such as liver-specific knockout (KO) mice, have been used in order to elucidate the hypoxia-mediated hepcidin regulation, however they exhibit overlaid adaptive responses difficult to interpret and may be developmentally lethal (Table 2). Additionally, several in vivo models of experimental ALD are described, mostly murine and rat models, however rodents differ from humans in terms of alcohol metabolism and do not develop severe cirrhosis [89].

Moreover, many in vivo and in vitro studies do not acknowledge the fact that the HIF1 kinetic is described as an HIF1–PHD feedback loop [86]. This feedback loop causes a permanent downregulation of HIF1 by PHD, a HIF1-controlled negative regulator of HIF. Thus, constant hypoxia will only transiently induce HIF1 while PHD upregulation eventually causes complete degradation of HIF. In other words, HIF1 only temporarily responds to oxygen lowering but not to absolute hypoxia per se [86]. For instance, despite low oxygen levels, HIF is usually not expressed in tissues and its expression has a low correlation with human cancers [101,102].

Therefore, the use of reliable in vitro models could be a valuable alternative to study hepcidin regulation by alcohol-induced hypoxia and H_2_O_2_ generation.

## 7. The GOX/CAT System Allows for Independent Study of Hypoxia and Hydrogen Peroxide

**Figure 4:**
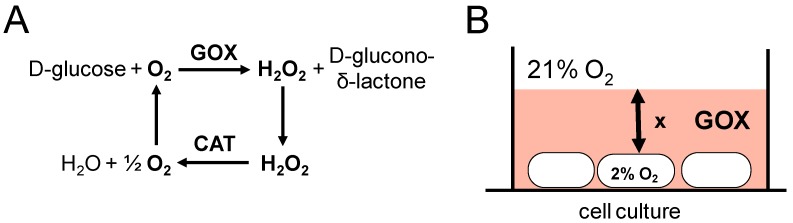
The principle of the glucose oxidase/catalase (GOX/CAT) system for independent control of hypoxia and H_2_O_2_ levels (adapted from [87]). (**A**) Stoichiometry of the GOX/CAT system. GOX converts 1 mol oxygen and glucose to 1 mol gluconolactone and H_2_O_2_, while CAT catalyzes the dismutation of 1 mol H_2_O_2_ into 0.5 mol oxygen and water. This results in net consumption of oxygen (hypoxia) and efficient control of H_2_O_2_. (**B**) The diffusion distance (x) from the medium surface to the bottom of the culture dish determines the degree of hypoxia besides the activity of oxygen-consuming GOX.

To overcome the limitations of the hypoxia chamber (delayed onset of hypoxia) and to study H_2_O_2_ signaling under hypoxic conditions, the GOX/CAT system has been recently developed [87]. GOX/CAT consists of glucose oxidase (GOX) and catalase (CAT), and allows the independent control and long-term maintenance of both hypoxia and H_2_O_2_ levels in cell culture (Figure 4A). GOX generates hypoxia by consuming the oxygen present in the culture medium depending on the oxygen diffusion distance to the adherent cells (Figure 4B). In contrast with the commonly used hypoxia chamber, the oxygen levels in the medium could be lowered within minutes at a defined rate. CAT activity controls the H_2_O_2_ concentration, allowing the generation of a wide range of sustained H_2_O_2_ levels over 24 h, from very low signaling levels to high toxic levels. Thus, the system can mimic the release of H_2_O_2_ by e.g., inflammatory cells or intracellular oxidases in a more realistic way than commonly used H_2_O_2_ bolus treatments. The independent control of hypoxia and low steady state concentrations of H_2_O_2_ makes the system suitable for studies of hypoxia or redox signaling.

Indeed, we successfully used the system to study HIF1α regulation, which responds to hypoxia and H_2_O_2_ [86]. Moreover, the GOX/CAT system has been previously applied in order to study signaling functions of H_2_O_2_ in iron homeostasis. We were able to describe the activation of IRP1 by exposure of cultured cells to sustained low levels of H_2_O_2_ [32,103,104]. As consequence of IRP1 activation several proteins involved in iron metabolism, such as TfR1, were post-transcriptionally upregulated [33,105]. A subsequent work also using the GOX/CAT system has demonstrated the H_2_O_2_-mediated induction of TfR1 also at the translational level [31]. Additionally, we have used the system to describe the transcriptional induction of hepcidin by sustained submicromolar H_2_O_2_ levels [58].

**Figure 5:**
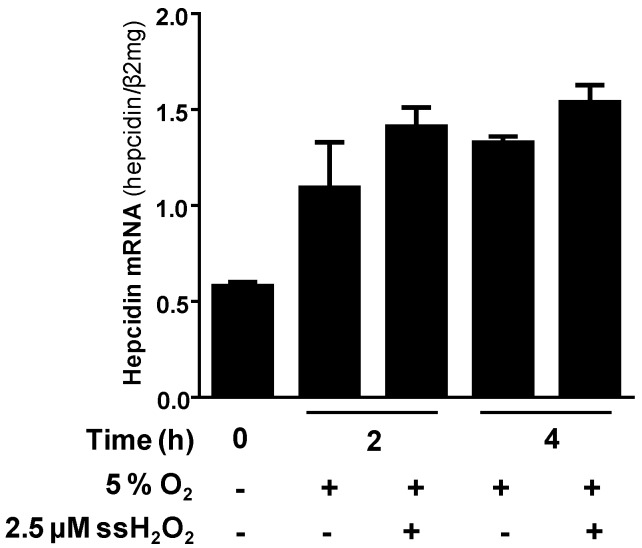
Hypoxia induces hepcidin alone or after co-exposure to low steady state (ss) levels of H_2_O_2_. Notably, hypoxia further enhances the H_2_O_2_-mediated upregulation of hepcidin via STAT3 [58]. Preliminary data indicate that hypoxia-associated hepcidin upregulation is also STAT3-mediated, pointing towards an oxidase as important upstream regulator of hepcidin. Hepcidin mRNA was quantified by quantitative real-time PCR and the results are represented as mean of hepcidin mRNA normalized to β2-microglobulin (β2mg) ± standard deviation.

Recently, we have analyzed, in vitro, the expression of hepcidin in response to physiologic hepatic oxygen levels (5% O_2_) combined with low levels of H_2_O_2_ applying the GOX/CAT system [106]. Surprisingly, we could show that hypoxia strongly enhanced the upregulation of hepcidin by low non-toxic H_2_O_2_ (Figure 5). Further detailed studies demonstrated that HIF1 was not involved in hepcidin induction by hypoxia but STAT3 (not shown). These findings let us to postulate that oxidases could be potential important upstream inducers of hepcidin since they produce peroxide by consuming oxygen. First preliminary data indicate that NOX4, an important NADPH oxidase expressed in hepatocytes, is indeed able to induce hepcidin. Thus, hypoxia induced NOX4 and the upregulation of NOX4 induced hepcidin [106].

**Figure 6:**
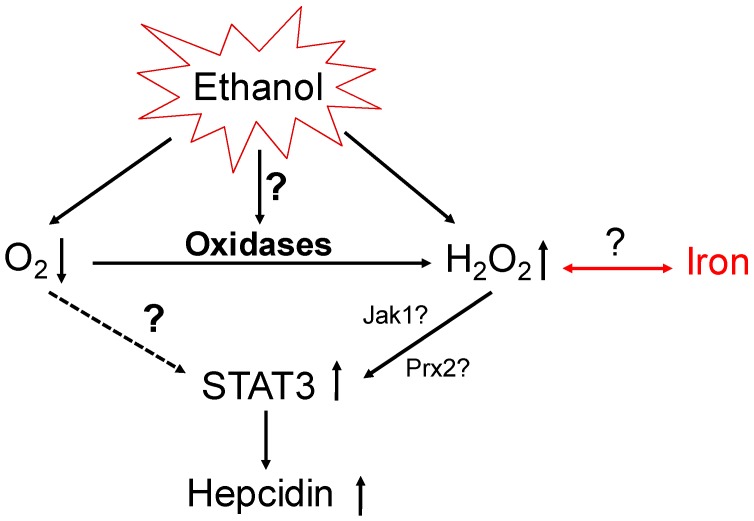
Role of oxidases in regulating hepcidin via hypoxia and H_2_O_2_ generation. The novel concept postulates that oxidases are involved in hepcidin regulation and alcohol-mediated hepatic iron overload. It remains to be studied whether this concept also explains iron-sensing of hepcidin (marked in red), which is still insufficiently understood. Jak1: Janus kinase 1.

The synergistic induction of hepcidin by hypoxia and H_2_O_2_ and the potential role of oxidases as important upstream regulators of hepcidin are schematically shown in Figure 6. Taken together, our recent novel and preliminary experimental insights suggest that hypoxia-mediated induction of hepcidin requires STAT3 and is enhanced by H_2_O_2_. Thus, peroxide-signaling might be the actual hypoxia sensitive pathway via oxidase. This concept will be discussed in more detail below.

## 8. Could NOX4 Be Responsible for Iron Overload in ALD?

Our preliminary data provide first evidence that oxidases such as NOX4 could be important upstream inducers of hepcidin. This could also explain why hepcidin is induced by hypoxia via STAT3 signaling which is known to be induced by H_2_O_2_ [58]. NADPH oxidases (NOXs) are multicomponent enzymes whose primary biological function is to produce ROS. NOXs are widely distributed through different tissues and cell types being involved in the transfer of electrons from NADPH to other molecules. NOX4 is mainly expressed in the liver and generates H_2_O_2_ in a highly regulated manner, since NOX4 is as vulnerable as other oxidases to growth factors, cytokines, or other stimuli. Additionally, NOX4 is the only NOX family enzyme constitutively active, dispensing the interaction with cytosolic regulators for its function; thus the activity of this oxidase only depends on its abundance [107].

Prior studies have described a hypoxia-mediated upregulation of NOX4 in lung tissue [108,109]. Regarding the liver, the co-localization of hypoxic, apoptotic, and NOX4-positive cells was already demonstrated in vivo, in a model of acute liver failure [110]. The authors concluded that hypoxia induced by the hepatic disorder leads to increased NOX4 expression, which caused increased oxidative stress and apoptosis [111]. Furthermore, induction of NOX4 by alcohol exposure was recently demonstrated in mice. Notably, the reversion of the alcohol-induced liver injury by administration of a NOX4 inhibitor highlighted its role in progression of ALD to HCC [110]. However, a specific role of NOX4 in hepatic iron accumulation has not been demonstrated so far.

**Figure 7:**
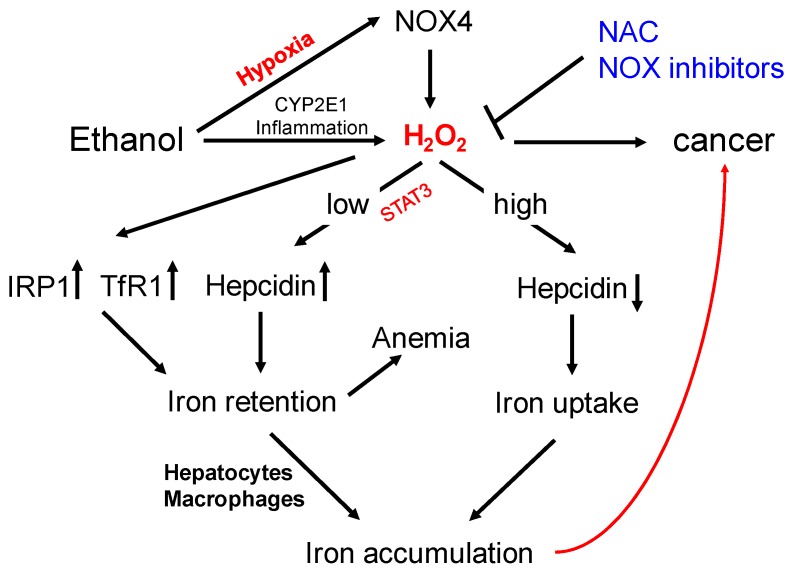
Scheme of molecular changes on iron metabolism mediated by alcohol and the role of hepcidin (adapted from [3]). Hypoxia and oxidases, such as NADPH oxidase 4 (NOX4), both contribute to H_2_O_2_-mediated hepcidin regulation. While low H_2_O_2_ levels could explain hepcidin induction via STAT3 and anemia, toxic levels cause suppression of hepcidin, ultimately leading to iron accumulation. The co-presence of hypoxia, reactive oxygen species, and iron are mandatory inducers of cancer. The novel concept may help in developing novel targeted therapies e.g. against NOX using NOX inhibitors. They could also explain the protective effects of i.v. N-acetylcysteine (NAC) infusions that seem to improve alcohol-induced liver damage.

Figure 7 updates our previous concept of iron overload in ALD [3] and incorporates novel data with regard to NOX4 and hypoxia. Thus, low peroxide and hypoxia cause hepcidin induction which could be responsible for the widely observed anemia in ALD patients. Under conditions of excess hypoxia and peroxide, however, hepcidin will be suppressed most likely due to unspecific inhibition of the transcription machinery [58]. These events would finally lead to iron overload and drastically increase the risk of developing HCC since the presence of ROS and iron is highly mutagenic. Interestingly, preliminary data from our laboratory suggest that other oxidases or an uncoupled respiratory chain can also induce hepcidin.

## 9. Conclusions and Future Directions

Hepatic iron overload, hypoxia, and increased oxidative stress are key features present in patients with ALD. Moreover, increasing clinical and experimental evidence suggest that hypoxia and hydrogen peroxide contribute to hepatic iron overload via dedicated molecular signaling on hepcidin through the STAT3 pathway. In the past decade, the lack of adequate models and multiple interpretational limitations have provided conflicting findings regarding hypoxia and ROS. The development of the GOX/CAT system allowed, for the first time, the independent control of the oxygen and H_2_O_2_ levels, simulating a typical environment found in most chronic liver diseases, namely ALD. Preliminary in vitro data indicate that hypoxia even enhances the peroxide-mediated upregulation of hepcidin in a concentration-dependent manner. These data point towards oxidases as important upstream regulators of hepcidin such as NOX4. Thus, NOX4 could represent a promising novel therapeutic target for treating alcohol-induced liver damage. NOX inhibitors could be potential therapeutic agents to prevent disease progression in selected patient cohorts. Moreover, the novel concept could provide a molecular rationale to better explain recent advances in treating ALD, e.g. with intravenous infusions of antioxidants such as N-acetylcysteine [112].

## Figures and Tables

**Table 1:** Hypoxia-mediated hepcidin regulation in hepatoma cell lines.

Cell Line	Conditions	Hepcidin mRNA	Suggested Mechanism	Reference
HepG2	1% O_2_; 24 or 48 h	↓	-	[63]
	10, 2 or 0.1% O_2_; 24 h	↓	-	[64]
	1% O_2_; 12 or 24 h	↓	Independent of HIF1	[77]
	1% O_2_; 16 h	↑	Independent of HIF1 and 2	[67]
Hep3B	0.5% O_2_; 48 h	↓	Inhibition of BMP/SMAD pathway by HIF-mediated induction of matriptase-2	[74]
Huh7	1% O_2_; 16 h	↑	Independent of HIF	[67]
	1% O_2_; 24 h	↑ ↓ ^1^	Hypoxia inhibits BMP/SMAD signaling pathway	[76]

^1^ Hypoxia-induced hepcidin mRNA in Huh7 monoculture but not in coculture with THP-1 macrophages. HIF: hypoxia-inducible factor; THP-1: human monocytic cell line.

**Table 2:** Genetically modified mouse models for the study of hepcidin regulation by hepatic hypoxia.

Mice Model	Survival	Implications for Iron Metabolism	Other effects	Reference
*Hif1α* KO ^1^	Not evaluated	No effect on hepcidin	-	[65]
*Hif2α* KO ^1^	Not evaluated	No effect on hepcidin	-	[69]
*Vhl* KO ^1^	5 to 7 weeks	Decreased hepatic iron, decreased hepcidin, increased IL-6 and IL-1β	Growth deficiency, alopecia, hepatomegaly and splenomegaly. Liver necrosis and steatosis	[65]
*Vhl/Hif1α* KO ^1^	3 to 5 weeks	Low serum iron, decreased hepcidin	Alopecia, weight loss, hepatomegaly and splenomegaly	[69]
*Hfe* KO	Not evaluated	Increases serum and hepatic iron, decreased splenic iron, decreased hepcidin	-	[90]
*Hjv* KO	Not decreased		Sterile males	[91]
*Tfr2* KO	Not evaluated		-	[92]
*Smad4* KO ^1^	Death during development		Weight loss, fibrosis, accumulation of neutrophiles and macrophages in the liver	[93]
*Bmp6* KO	Not evaluated		Delay in bone formation	[94]
*Tmprss6* KO (matriptase-2)	Not evaluated	Anemia, low iron stores, iron accumulation in enterocytes, increased hepcidin	Growth retardation. alopecia	[95]
*Stat3* KO ^1^	Not evaluated ^2^	Increased hepcidin without developing anemia	Insulin resistance, increased gluconeogenesis, higher susceptibility to hepatic damaged	[96,97,98,99]
*Prx2* KO	Not evaluated	No significant changes in hepcidin expression	Cardiovascular disease, splenomegaly, hemolytic anemia, inflammation, decreased immune function	[60,100]

^1^ Hepatocyte-specific genetically modified model; ^2^ Total STAT3 knockout (KO) mice that die during embryogenesis. BMP6: Bone morphogenic protein 6; HJV: Hemojuvelin; Prx2: Peroxiredoxin 2; SMAD4: Suppressor of mothers against decapentaplegic homolog 4; STAT3: Signal transducer and activator of transcription 3; TFR2: Transferrin receptor 2; VHL: Von Hippel-Lindau protein.
